# Impact of water deficit stress in maize: Phenology and yield components

**DOI:** 10.1038/s41598-020-59689-7

**Published:** 2020-02-19

**Authors:** R. P. Sah, M. Chakraborty, K. Prasad, M. Pandit, V. K. Tudu, M. K. Chakravarty, S. C. Narayan, M. Rana, D. Moharana

**Affiliations:** 1 Present Address: ICAR-National Rice Research Institute, Cuttack, Odisha India; 2 Department of Genetics and Plant Breeding, Birsa Agricultural University, Kanke, Ranchi, Jharkhand India; 3 Department of Entomology, Birsa Agricultural University, Kanke, Ranchi, Jharkhand India; 4 Indian Grassland and Fodder Research Institute, Jhansi, Uttar Pradesh India; 5 ICAR-National Rice Research Institute, Cuttack, Odisha India

**Keywords:** Plant breeding, Environmental impact

## Abstract

Fifteen million farmers in India engaged in Maize cultivation. India would require 45 MMT of Maize by 2022. But, only 15% of cultivated area of maize is under irrigation and water shortage has been a challenge for sustainability of maize production. Water deficit stress (WDS) during pre-flowering and grain filling stages massively affects the plant performance due to imprecise traits function. Thus, the effect of WDS on non-drought tolerant (NDT) and drought tolerant (DT) maize lines were investigated. WDS increased the flowering days, days to maturity, anthesis silk interval, decreased the leaf number, abnormal expression of secondary stress responsive traits, loss of normal root architecture which overall lead to a reduction in GY/ha. WDS at flowering and grain filling stage leads to significant yield penalty especially in NDT lines than DT lines. The yield penalty was ranged from 34.28 to 66.15% in NDT and 38.48 to 55.95% in DT lines due to WDS. Using multiple statistics, traits which improve WDS tolerance in maize were identified viz; number of leaves, number of stomata on lower surface of leaf, leaf angle at ear forming node internodal length between 3^rd^ and 4^th^ leaf from top, flag leaf length, flag leaf width, ear per plants, leaf senescence, pollen stainability, root fresh weight and root length. These traits would help in trait specific breeding in maize for WDS tolerance.

## Introduction

Maize (*Zea mays* L.) is a multipurpose crop with wide adaptability to different agro-climatic conditions. It is grown in most parts of the world, up to 3000 m above mean sea level^1^.This crop is preferred by farmers due to its grain production potential being the highest among cereals^2^, its dual-purpose use (grain and fodder)^3,4^; used as a cash crop (specialty corn: green ear, baby corn, sweet corn and popcorn)^5^, and raw materials for industry.

In fact, maize is not a food crop but an industrial crop, as only 12–13% of its production is used for human consumption globally^6^. It is cultivated in an area of nearly 150 Mha in approximately 160 countries, which constitutes 36% (782 Mt) of the global grain production^7^. Of the total maize grain produced in the world, approximately 70–80% is used as feed, whereas in India, approximately 49–51% is used as poultry feed, 12% is used as animal feed, and 25% is used for human consumption^8^. The maize cob powder is used as fillers for explosives in the manufacture of plastics, glues, adhesives, resin, vinegar and artificial leather. It is often a part of diluents and carrier in insecticides and pesticides formulation, and also used for pulp, paper and hard boards manufacturing. Grain is used for commercial starch extraction, corn flour, corn oil extraction, corn flakes and corn syrup preparation.

In many countries, maize is grown in areas that receive 300–500 mm of precipitation, which is near or below the critical level for obtaining a good yield^9,10^. India has a wide diversity of agro-climates, where maize is produce due to its highly adaptable nature^1^. In India, maize requires 500 to 600 mm of rainfall for at least good production, but production also depends on the duration of the variety. Approximately 80% of wet-season maize areas are rain-fed, where crops are susceptible to the erratic behavior of rains^11^. The rainfall mostly occurs in the early growth stages, and the crop faces water deficit stress (WDS) from the pre-flowering to late grain-filling stages. Such problems considerably affect the phenotype, reproductive system and seed set. Hence, maize production in the wet season and in rain-fed regions is declining due to natural intermittent WDS. However, dry-season maize areas are currently expanding in India due to higher productivity than in wet-season areas and are providing good-quality fodder in either the dry or green (stay-green) form for livestock. In the last 25–30 years, WDS usually occurred during the months of August to September in the wet season and after the 1^st^ fortnight of February in the dry season at the experimental location (Supplementary Fig. 1). In both periods, enough water is usually available to allow the plant to grow to the vegetative stage. Afterwards, from pre-flowering to grain filling, plants are severely affected by a prolonged rainfall interval in the wet season and a water shortage in the dry season.

Dry-season maize crops exhibit late maturity due to a prolonged cold period at the early crop growth stage between the seedling and knee-high stage. Consequently, a longer duration requires a greater water input. Hence, loss of grain production in the dry season is connected to shortages of water used for irrigation^12^. The loss of yield varies from 30–90% depending on the crop stage and the degree and duration of WDS^13^. The stages of maize susceptible to WDS are the vegetative, silking (flowering) and ear stages (grain filling), where yield loss may be as high as 25%, 50%, and 21%, respectively^14^. Thus, based on the prevailing weather in eastern India, WDS was imposed at 2 critical stages, i.e., the flowering and grain-filling stages, in our experiment.

The loss of phenotypic expression under WDS is obvious in most cereals. In maize, phenotypic expression is also suppressed after a critical level of WDS. The effect is prominent, such as a reduction in the green-leaf duration (stay-green), plant performance^1^, ear length, seed weight^15^, plant height^16^, number of grains per ear, leaf number^1,17,18^, ears per plant, kernel rows per ear, kernels per row, and early leaf senescence, even during flowering^16,19^. Stay-green genotypes are often superior to non-stay-green ones, especially under water deficit conditions, and are often correlated with grain-yield traits (grain yield, ear length, and kernels per row). Increases in below ground traits such as root mass and rooting depth increase the plant’s ability to cope with drought stress^20–22^. Hence, such traits are also considered important for identifying potential parents for hybrid development^23^. However, phenotypic expression is not always suppressed under WDS. In some cases, traits are overexpressed in certain genotypes. This overexpression depends on the nature of the traits and individual genotypic backgrounds. Thus, both loss and gain of phenotypic expression were precisely measured and considered in the present experiment.

The performance of any maize line depends on its genetic constitution and the response of the desirable trait under stress and non-stress conditions. WDS-tolerant lines were developed, and affected traits were considered as selection criteria for parental selection. However, their performance is best for a particular WDS level, and they fail to perform well under even a small change in WDS. Practically, WDS is not constant throughout the cropping period; it changes continuously depending on the crop stage, amount of available moisture and soil type. Such conditions are prevalent in the majority of experiments. Thus, proper phenotyping for the identification of key phenological traits associated with yield improvement remains a major area of research. A comparative analysis of phenotypic expression under WDS is also not available. Thus, in the present experiment, 7 levels of WDS were maintained to simulate the environmental variability of eastern India. Under these conditions, a new source of maize lines was developed and evaluated under multiple WDS levels (prevailing under wet and dry seasons in the eastern Indian region) to determine the (i) performance of maize lines under WDS conditions, (ii) range of loss and gain of phenotypic expression (LGP) under WDS, (iii) relationship between grain yield and phenological traits and (iv) effective phenotype conferring WDS tolerance in maize. The majority of the maize in eastern India is grown under rain-fed conditions, and marginal farmers in this region are unable to practice crop management strategies that might mitigate these constraints. In such situations, breeding for WDS-tolerant maize remains the best alternative. The results obtained here will help develop strategies for trait-specific breeding to enable maize improvement under WDS conditions.

## Materials and Methods

### Land preparation and cultural practices

The experiments were conducted in the experimental field and rainout shelter available at the Faculty of Agriculture, Birsa Agricultural University (BAU), Ranchi, Jharkhand, India. The field was cleaned, well plowed, and cleared of debris; it was demarcated with lines and pegs and leveled using a hoe before seeds of the genotypes were sown. A pre-emergence weed control chemical, i.e., atrazine, was used at a concentration of 1 kg a.i./ha. The other cultural practices and fertilization were followed as per recommendations.

### Genetic material and evaluation

Eleven maize lines were used for the present experiment, 6 of which (BAUIM-1, BAUIM-2, BAUIM-3, BAUIM-4, BQPM-4 and BAUIM-5) were developed by BAU, Ranchi; 2 of which (CM-500 and CM-111) were developed by the Indian Institute of Maize Research (IIMR), Ludhiana; and 3 of which (HKI-1532, HKI-335 and HKI-488) were developed by Chaudhary Charan Singh Agricultural University (CCSHAU), Haryana. The 3 lines developed by CCSHAU were drought tolerant (DT). These 3 lines were pre-evaluated in 2010–2011 at BAU, Ranchi, to confirm their performance under the WDS (50 kPa) used in our experiment. For both drought tolerant (DT) and non-drought tolerant (NDT) maize lines, the effects of irrigation regimes on yield, growth parameters and physiological parameters were analyzed using a randomized complete block design with three replicates. All the seeds of the maize lines were obtained from ICAR-All India Coordinated Research Projects-Maize, Ranchi Center.

### Managed stress environment and irrigation

In the wet season of 2013, 3 irrigation regimes and in dry season 2014, 4 irrigation regimes were created and maintained. For the irrigated condition, the tensiometer was maintained at −30 kPa (considered as standard). Reading above −30 kPa was considered as water deficit stress (WDS); accordingly, as per the environmental variability of the region, different stress levels were created for evaluation. The details of the water management used to maintain the WDS levels are presented in Table 1. The irrigation water was applied as per tensiometer reading placed at root-zone depth in each irrigated or WDS level. The locations selected for the tensiometer were representative of the general conditions of field.

**Table 1:** Irrigation regimes used for evaluation of NDT and DT maize inbred lines.

Seasons	Evaluation	Crop stage
**Wet season-2013**		
Irrigated	Field	No stress, Irrigation at −30 kPa
Rainfed	Field	Stress observed during 30–35 DAS and reproductive stage
Light stress	Rainout shelter	Stress imposed during flowering and grain filling, irrigation at −50 kPa
**Dry season-2013–14**		
Irrigated	Field	No stress, Irrigation at −30 kPa
Mild to mild severe	Field	Mild stress (−40 kPa) at flowering and mild severe (−60 kPa) stress during grain filling
Mild severe to severe	Rainout shelter	Mild severe (−60 kPa) stress at flowering and severe (−80 kPa) stress during grain filling
Severe stress	Rainout shelter	Severe (−80 kPa) stress during flowering and grain filling both.

No stress was imposed up to knee high stage to obtain uniform plant population and growth.

### Data collection and statistical analysis

Phenotypic data were recorded on 38 quantitative traits and grouped in to 6 categories viz; (i) Flowering and maturity: days to 50% tasseling (DA), days to 50% silking (DS), anthesis silk interval (ASI) in days and days to 75% dry husk (DDH); (ii) Vegetative and leaf traits: plant height (PH) in cm, number of leaves per plant (NL), number of stomata on upper surface of leaf (SU), number of stomata on lower surface of leaf (SL), 3^rd^ leaf angle from top (LA-3), leaf angle at ear forming node (LA-C), internodal length between 3^rd^ and 4^th^ leaf from top (INL 3–4) in cm, flag leaf length (FLL) in cm and flag leaf width (FLW) in cm; (iii) Ear traits: ear height (EH) in cm, ear length (EL) in cm, ear width (EW) in cm and number of husk per ear (H/C); (iv) Root traits: number of brace root per plant (NBR), root fresh weight (RFW) in grams, root dry weight (RDW) in grams, root length (RL) in cm, root volume (RV) in cc and number of roots >10 cm (RN); (v) Yield traits and stress indices: number of ear per plant (C/P), number of kernels per rows (K/R), number of kernel rows per ear (KR/C), 1000 seed weight (SW) in grams, grain yield per hectare (GY/ha) in quintals, modified stress tolerance index (KiSTI) and yield index (YI); (vi) Secondary stress responsive traits (SSRT): relative leaf water content (RLWS) in percentage, stay green (SG) in percentage, leaf senescence (LS) score, leaf rolling (LR) score, leaf firing (LF) in score, pollen stainability in percentage (PS%), tassel blast (TB) in percentage, and bareness percentage (BP). The collected data were statistically analyzed following analysis of variance, principal component analysis (PCA) and co-heritability (h^2^) using Indo-Stat 7.5 software (Indo-stat, Hyderabad, India). The Microsoft Excel 2016 was used for regression analysis, preparation regression curve, and t-test at 5% level of significance.

## Results

### Phenotypic variation of NDT and DT maize lines

Non-drought tolerant (NDT) lines generally perform better in a favorable environment (irrigated), whereas DT lines are intentionally developed to perform better under unfavorable (WDS) conditions. The differences in these two types of lines are mostly genetic and lead to distinct phenotypic differences when the lines are grown in target environments. Thus, in the present experiment, 6 important groups of 38 traits were phenotyped carefully. The average magnitude of phenotypic change is presented in parentheses and the traits at least ±15% effect and score of >3 due to WDS were only discussed for easy understanding.

#### Performance of NDT maize lines under WDS conditions

An increase in the mean value of DA (9.81%), DS (14.33%) and ASI (86.44%) was observed under WDS compared to irrigated conditions. High value increased the flowering duration and extended the DDH by 11.98%. Thus, there was a negative impact on flowering and maturity traits. Similarly, the mean value of PH (18.24%), INL3–4 (17.99%), FLW (18.00%), EH (19.08%), EL (36.07%), EW (27.68), C/P (18.87%), K/R (36.72%), KR/C (29.55%), GY/ha (51.49%), KiSTI (16.67%), NBR (51.06%), RFW (38.87%), RDW (26.01%), RL (39.79%) and RV (43.14%) decreased under WDS. This decrease in the mean value of the traits reflects a reduction in the performance of the plants in terms of vegetative growth, root and yield attributes. The mean value of SSRT under WDS compared to irrigated conditions was lower in RLWC (15.36%) and PS (52.58). In contrast, it was higher in LS (score of 2.21), LR (score of 3.93), TB (score of 3.42) and BP (21.88%), as is usually expected to for these traits. Thus, the NDT lines were significantly affected by and showed a marked difference (% difference) in trait expression under WDS (Tables 2 and 3).

**Table 2:** Variation and effect on 38 traits of NDT lines under WDS.

Traits	Mean I	WDS	Change due to WDS	Range I	WDS	LGP-WDS	Gain
						Loss	
**Flowering and maturity**						% (except days and score)	% (except days and score)
DA	81.22	89.19	7.97 (9.81)	77.00–86.00	84.38–95.38	**—**	4.13–11.88 Days
DS	86.31	98.68	12.37 (14.33)	82.00–91.50	91.88–103.50	—	9.88–16.00 Days
ASI	5.09	9.49	4.40 (86.44)	4.25–6.00	7.50–11.25	—	2.50–7.00 Days
DDH	113.34	126.92	13.58 (11.98)	110.25–116.50	121.00–131.00	—	10.75–17.00 Days
**Vegetative stage and leaf traits**							
PH	127.81	104.50	−23.31 (18.24)	101.25–157.67	88.81–130.07	9.34–28.72	—
NL	11.85	10.97	−0.88 (7.43)	10.44–13.93	9.14–13.10	14.10	5.99
SU	113.17	99.65	−13.52 (11.95)	81.00–140.83	83.42–118.92	37.42	39.90
SL	154.40	138.06	−16.34 (10.58)	124.00–183.83	123.47–163.24	30.69	1.63
LA-3	27.15	24.56	−2.59 (9.54)	18.73–33.21	21.46–28.21	24.72	20.82
LA-C	30.22	27.12	−3.10 (10.26)	24.72–34.07	23.45–32.07	20.30	0.04
INL3–4	11.17	9.16	−2.01 (17.99)	8.75–12.60	6.63–10.87	29.47	11.74
FLL	27.89	24.48	−3.41 (12.23)	20.50–34.88	18.28–32.36	33.98	10.29
FLW	4.39	3.60	−0.79 (18.00)	3.63–5.01	3.27–4.04	29.47	7.71
**Ear traits**							
EH	59.89	48.46	−11.43 (19.08)	41.42–80.17	34.12–65.08	4.86–38.05	—
EL	16.58	10.60	−5.98 (36.07)	12.88–21.06	8.35–12.50	28.13–50.13	—
EW	4.95	3.58	−1.37 (27.68)	4.65–5.40	3.07–4.09	16.22–35.65	—
H/C	7.96	8.45	0.49 (6.16)	7.09–8.75	7.98–8.92	5.49	18.44
**Yield and stress indices**							
CP	1.06	0.86	−0.20 (18.87)	0.95–1.19	0.74–1.06	0.70–36.36	—
K/R	24.48	15.49	−8.99 (36.72)	20.51–29.05	12.91–19.72	16.26–51.88	—
KR/C	13.57	9.56	−4.01 (29.55)	12.17–14.63	7.33–11.83	16.67–47.30	—
SW	228.79	208.52	−20.27 (8.86)	170.75–263.19	188.87–248.45	21.60	24.35
GY/ha	32.12	15.58	−16.54 (51.49)	27.31–37.49	12.23–21.84	34.28–66.15	—
KiSTI	0.30	0.25	−0.05 (16.67)	0.22–0.39	0.16–0.37	59.62	21.31
YI	0.54	0.47	−0.07 (12.96)	0.47–0.63	0.36–0.60	42.80	9.55
**Stress responsive traits**							
RLWC	93.52	78.16	−15.36 (16.42)	89.13–95.16	73.66–82.45	12.05–20.96	—
SG	15.08	12.80	−2.28 (15.12)	6.41–47.32	5.99–30.86	44.39	46.49
LS	2.40	4.61	2.21 (92.08)	2.00–3.17	3.88–5.79	—	1.71–3.46 Score
LR	1.36	5.29	3.93 (288.97)	0.88–1.75	4.36–6.34	—	2.96–4.67 Score
LF	0.64	2.45	1.81 (282.81)	0.48–0.85	1.72–3.15	—	1.05–2.50 Score
PS	98.63	46.05	−52.58 (53.31)	98.22–99.45	44.01–48.79	49.65–55.43	—
TB	1.48	4.90	3.42 (231.08)	1.00–3.17	4.05–6.17	—	2.39–5.17 Score
BP	1.10	22.98	21.88 (1989.09)	0.61–1.42	14.66–36.22	—	13.52–34.80
**Root traits**							
NBR	1.41	0.69	−0.72 (51.06)	0.50–2.00	0.25–1.17	8.50–72.17	—
RFW	29.84	18.24	−11.6 (38.87)	22.62–45.86	8.79–27.99	29.24–61.36	—
RDW	8.65	6.40	−2.25 (26.01)	4.53–18.83	2.03–11.13	55.19	3.98
RL	26.84	16.16	−10.68 (39.79)	20.75–31.50	13.83–20.04	26.45–48.36	—
RV	21.88	12.44	−9.44 (43.14)	15.75–33.75	9.39–16.94	13.63–60.20	—
RN	39.47	33.88	−5.59 (14.16)	25.25–49.50	21.57–48.50	39.94	8.15

Data in parenthesis is the percentage change; I: Irrigated; WDS: Water Deficit Stress; LGP-WDS: Loss and grain in phenotype under WDS; DT: Drought tolerant; NDT: Non-drought tolerant; SSRT: Secondary stress responsive traits; DA: Days to 50% tasseling; DS: Days to 50% silking; ASI: Anthesis silk interval; DDH: Days to 75% dry husk; PH: Plant height; NL: Number of leaves per plant; SU: Number of stomata on upper surface of leaf; SL: Number of stomata on lower surface of leaf; LA-3: 3rd leaf angle from top; LA-C: Leaf angle at ear forming node; INL3–4: Internodal length between 3rd and 4th leaf from top; FLL: Flag leaf length; FLW: Flag leaf width; EH: Ear height; EL: Ear length; EW: Ear width; H/C: Number of husk per ear; C/P: Number of ear per plant; K/R: Number of kernels per rows; KR/C: Number of kernel rows per ear; SW: 1000 seed weight; GY/ha: Grain yield per hectare; KiSTI: Modified stress tolerance index; YI: Yield index; RLWC: Relative leaf water content; SG: Stay green; LS: Leaf senescence; LR: Leaf rolling; LF: Leaf firing; PS%: Pollen stainability; TB: Tassel blast and BP: Bareness percentage; NBR: Number of brace root per plant; RFW: Root fresh weight; RDW: Root dry weight; RL: Root length; RV: Root volume; RN: Number of roots >10 cm.

**Table 3:** Variation and effect on 38 traits of DT lines under WDS.

Traits	Mean I	WDS	Change due to WDS	Range I	WDS	LG-WDS	
						Loss	Gain
**Flowering and maturity**				% (except days and score)	% (except days and score)		
DA	79.50	87.46	7.96 (10.01)	74.25–84.75	84.50–89.63	—	3.50–10.25 Days
DS	83.17	95.38	12.21 (14.68)	78.25–87.75	93.63–97.00	—	7.75–15.38 Days
ASI	3.67	7.92	4.25 (115.80)	3.00–4.00	7.25–9.13	—	3.38–5.13 Days
DDH	115.17	127.38	12.21 (10.60)	114.25–115.75	123.63–130.75	—	9.38–15.25 Days
**Vegetative stage and leaf traits**							
PH	130.63	105.52	−25.11 (19.22)	113.96–160.92	86.46–127.15	12.00–24.13	—
NL	12.03	11.08	−0.95 (7.90)	11.63–12.33	10.20–11.63	4.08–12.30	—
SU	117.50	105.64	−11.86 (10.09)	93.67–130.33	90.67–114.25	29.44	21.98
SL	146.11	134.42	−11.69 (8.00)	143.00–148.00	113.72–153.88	22.82	7.61
LA-3	29.18	26.61	−2.57 (8.81)	26.17–32.86	24.64–28.45	25.03	2.27
LA-C	32.49	28.05	−4.44 (13.67)	24.40–37.07	25.79–31.26	26.88	5.69
INL3–4	11.80	9.90	−1.90 (16.1)	10.91–12.50	7.86–11.14	10.86–27.92	—
FLL	30.13	21.61	−8.52 (28.28)	29.56–30.84	19.63–24.30	19.00–36.35	—
FLW	4.41	3.01	−1.40 (31.75)	4.04–4.60	2.54–3.26	19.43–44.61	—
**Ear traits**							
EH	63.33	46.84	−16.49 (26.04)	47.67–77.50	37.38–58.75	21.58–31.54	—
EL	15.72	11.00	−4.72 (30.03)	11.99–18.70	9.67–12.49	19.37–34.17	—
EW	4.83	3.27	−1.56 (32.3)	4.37–5.29	2.88–3.71	26.00–40.41	—
H/C	8.69	8.70	0.01 (0.12)	8.17–9.00	7.69–10.15	14.58	24.28
**Yield and stress index**							
CP	1.09	0.91	−0.18 (16.51)	0.97–1.26	0.83–0.99	12.32–20.92	—
K/R	24.54	17.20	−7.34 (29.91)	21.38–27.85	15.88–19.29	25.73–32.63	—
KR/C	13.34	9.38	−3.96 (29.69)	12.96–13.92	7.57–10.69	18.69–45.62	—
SW	250.39	206.14	−44.25 (17.67)	246.18–258.38	200.42–212.13	16.37–18.73	—
GY/ha	35.53	18.28	−17.25 (48.55)	27.76–45.27	14.26–20.65	38.48–55.95	—
KiSTI	0.38	0.37	−0.01 (2.63)	0.24–0.57	0.25–0.43	23.89	25.00
YI	0.60	0.58	−0.02 (3.33)	0.48–0.75	0.49–0.63	17.00	11.95
**Stress responsive traits**							
RLWC	93.70	75.05	−18.65 (19.90)	92.45–94.64	71.97–77.14	18.49–22.15	—
SG	51.38	35.03	−16.35 (31.82)	8.66–78.91	7.34–53.50	15.16–43.93	—
LS	2.50	4.89	2.39 (95.60)	2.00–3.00	4.00–5.71	—	1.50–2.96 Score
LR	1.65	4.70	3.05 (184.85)	1.42–1.91	4.34–5.40	—	2.43–3.78 Score
LF	0.56	2.65	2.09 (373.21)	0.30–0.70	2.06–3.30	—	1.77–2.62 Score
PS	98.95	46.77	−52.18 (52.73)	98.44–99.77	45.47–47.47	51.06–54.30	—
TB	1.00	4.37	3.37 (337.00)	1.00–1.00	3.84–5.15	—	2.84–4.15 Score
BP	0.67	26.04	25.37 (3786.57)	0.54–0.85	19.54–31.03	—	18.69–30.49
**Root traits**							
NBR	1.28	0.69	−0.59 (46.09)	0.84–2.00	0.58–0.83	20.36–58.38	—
RFW	27.56	13.78	−13.78 (50.00)	21.90–30.41	9.28–17.77	41.55–57.63	—
RDW	9.52	4.69	−4.83 (50.74)	7.97–11.09	2.97–6.78	38.81–62.70	—
RL	30.11	16.51	−13.60 (45.17)	27.42–32.09	15.17–17.80	44.53–46.25	—
RV	20.10	14.74	−5.36 (26.67)	15.68–23.13	12.83–18.11	15.22–40.34	—
RN	39.97	27.73	−12.24 (30.62)	31.59–50.09	23.44–31.07	9.17–38.73	—

Data in parenthesis is the percentage change; I: Irrigated; WDS: Water Deficit Stress; LGP-WDS: Loss and grain in phenotype under WDS; DT: Drought tolerant; NDT: Non-drought tolerant; DA: Days to 50% tasseling; DS: Days to 50% silking; ASI: Anthesis silk interval; DDH: Days to 75% dry husk; PH: Plant height; NL: Number of leaves per plant; SU: Number of stomata on upper surface of leaf; SL: Number of stomata on lower surface of leaf; LA-3: 3rd leaf angle from top; LA-C: Leaf angle at ear forming node; INL3–4: Internodal length between 3rd and 4th leaf from top; FLL: Flag leaf length; FLW: Flag leaf width; EH: Ear height; EL: Ear length; EW: Ear width; H/C: Number of husk per ear; C/P: Number of ear per plant; K/R: Number of kernels per rows; KR/C: Number of kernel rows per ear; SW: 1000 seed weight; GY/ha: Grain yield per hectare; KiSTI: Modified stress tolerance index; YI: Yield index; RLWC: Relative leaf water content; SG: Stay green; LS: Leaf senescence; LR: Leaf rolling; LF: Leaf firing; PS%: Pollen stainability; TB: Tassel blast and BP: Bareness percentage; NBR: Number of brace root per plant; RFW: Root fresh weight; RDW: Root dry weight; RL: Root length; RV: Root volume; RN: Number of roots >10 cm.

#### Performance of DT maize lines under WDS conditions

In the DT maize lines, the mean values of ASI (115.80%) was high under WDS than under irrigated conditions. Overall, higher means of flowering traits led to an extension of DDH by 10.60% (Tables 2 and 3). Similarly, the mean values of following traits were lower under WDS viz; PH (19.22%), INL3–4 (16.10%), FLL (28.28%), FLW (31.75%), EH (26.04%), EL (30.03%), EW (32.30%), C/P (16.51%), K/R (29.91%), KR/C (29.69%), SW (17.67%), GY/ha (48.55%), NBR (46.09%), RFW (50.00%), RDW (50.74%), RL (45.17%), RV (26.67%) and RN (30.62%). These decreases in the mean value of the traits reduced the expression of phenotypes related to vegetative growth, roots and yield. The SSRT also showed a lower mean value under WDS than under irrigated conditions, including RLWC (19.90%), SG (31.82%) and PS (52.73%). However, this mean value was higher under WDS than under irrigated conditions for LR (score of 3.05), TB (score of 3.37) and BP (25.378%) (Table 3).

The variation in the range (minimum and maximum values) for DA, DS, CP, SG and LF was higher in DT than in NDT lines under irrigated conditions. However, under WDS conditions, ASI, DDH, H/C, C/P, K/R, RLWC and SG were lower in DT than in NDT lines, which is an ideal response under WDS (Tables 2 and 3).

### Effect of WDS on LGP

The performance of both the NDT and DT maize lines declined under WDS compared to irrigated conditions. The mean value as well as the range of 38 traits was lower under WDS. However, 10 traits (DA, DS, ASI, DDH, H/C, LS, LR, LF, TB and BP) had a higher mean and range in both the NDT and DT lines. These observations were based on the mean of the traits. In addition, another type of estimate was obtained, i.e., the response of individual lines to WDS conditions (Supplementary Table 1). The response of individual lines was different for each trait, which was similar to findings described on the basis of the mean value. The mean value was actually the average performance of a group of genotypes, and expression of a trait under WDS was be reduced in one line but increased in another line.

### Relationship between yield and phenological traits

**Figure 1:**
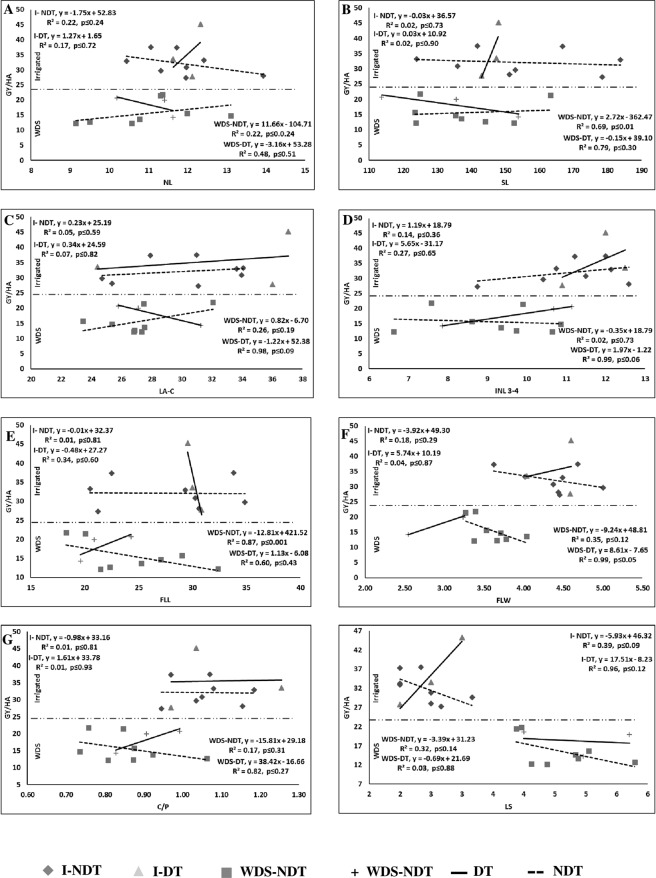
Relationship of different traits with grain yield (GY/ha) under irrigated and water deficit stress (WDS) condition. NL: Number of leaves per plant (**A**) SL: Number of stomata on lower surface of leaf (**B**) LA-C: Leaf angle at ear forming (**C**) INL3-4: Internodal length between 3rd and 4th leaf from top (**D**) FLL: Flag leaf length (**E**) FLW: Flag leaf width (**F**) C/P: Number of ear per plant (**G**) LF: Leaf senescence (**H**) I: Evaluated under irrigated condition; WDS: Evaluated under WDS condition; DT: Drought tolerant; NDT: Non-drought tolerant.

**Figure 2:**
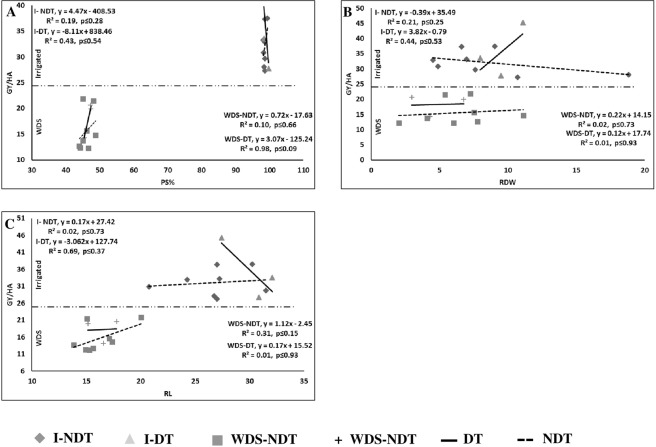
Relationship of different traits with grain yield (GY/ha) under irrigated and water deficit stress (WDS) condition. PD: Pollen stainability (**A**) RDW: Root dry weight (**B**) RL: Root length (**C**) WDS: Evaluated under WDS condition; DT: Drought tolerant; NDT: Non-drought tolerant.

Grain yield is the ultimate indicator of the economic value of a maize line. It is a complex trait and has an association with numerous other traits. Thus, graphical representation using linear regression analysis of the relationship between grain yield and selected traits is presented in Figs. 1 and 2. The traits identified for WDS tolerance only presented in graphical format. A trait was considered relevant if it exhibited R^2^ ≥ 0.40 because p-value obviously high for smaller number of sample (8 lines in NDT and 3 lines in DT).

#### Flowering and maturity

Maize is a geitonogamous species, where synchronization of DA and DS is essential to reduce ASI and a higher abundance of pollen during stigma receptivity. After survival of the plants, flowering traits are the second most important morphological parameter considered for grain setting under WDS. A linear relationship of grain yield with flowering traits and maturity was detected. The irrigated non-drought tolerant (I-NDT) lines exhibited weak relationships between all flowering and maturity traits with GY/ha. However, in the water deficit stress non -drought tolerant (WDS-NDT) lines, DA (R^2^ = 0.53) and DS (R^2^ = 0.43) had a strong relationship with GY/ha. Similarly, in the I-DT lines, ASI (R^2^ = 0.57) had a strong relationship with GY/ha, whereas in the WDS-DT lines, ASI (R^2^ = 0.40) and DDH (R^2^ = 0.76) had a strong relationship with GY/ha. Both types of lines showed distinct relationships with GY/ha when grown under WDS.

#### Vegetative and leaf traits

Plant height improves the crop canopy and surface area. The internodal length and height are highly correlated parameters. Each internode is borne on a leaf and generally appears after 13 leaf tassels are produced. The internal water and temperature balance in plants is regulated by stomata, whose efficiency depends on their frequency. I-NDT exhibited no strong relationships with height, leaf or internode traits. However, the WDS-NDT lines had a strong and positive relationship with GY/ha in PH (R^2^ = 0.93), SU (R^2^ = 0.93), SL (R^2^ = 0.69), LA-3 (R^2^ = 0.60), and FLL (R^2^ = 0.87). In the case of I-DT, only SU (R^2^ = 0.51) had a strong relationship with GY/ha. However, in the WDS-DT lines, PH (R^2^ = 0.55), NL (R^2^ = 0.48), SU (R^2^ = 0.43), SL (R^2^ = 0.79), LA-3 (R^2^ = 0.59), LA-C (R^2^ = 0.98), INL3–4 (R^2^ = 0.99), FLL (R^2^ = 0.60) and FLW (R^2^ = 0.99) had a strong relationship with GY/ha (Fig. 1A–F).

#### Ear traits

The ear of the plant contains the female inflorescence, where grains are set. Ear traits such as EH, EL, EW and H/C are the major morphological traits of ears. A medium ear height helps the ear receive a large number of pollen grains for fertilization and reduces the incidence of animal damage. I-NDT had a weak relationship with GY/ha for all ear traits; however, in WDS-NDT, this relationship with GY/ha was strong and positive for EL (R^2^ = 0.49). In the I-DT lines, all 3 traits, namely, EL (R^2^ = 0.86), EW (R^2^ = 0.96), and H/C (R^2^ = 0.6), had strong and positive relationships with GY/ha. However, in the WDS-DT lines, these relationships were observed only for EL (R^2^ = 0.56) and H/C (R^2^ = 0.89).

#### Root traits

The root structure of maize plays a major role in lodging, the uptake of nutrients and water and survival under unfavorable soil conditions. Six root traits were measured in the present experiment: NBR, RFW, RDW, RL, RV and RN. I-NDT and WDS-DT lines had a strong and negative relationship with GY/ha only in RV (I-NDT, R^2^ = −0.69; WDS-DT, R^2^ = −0.98), whereas in WDS-NDT, a weak relationship was observed for all the root traits. In the I-DT lines, NBR (R^2^ = −0.96), RDW (R^2^ = −0.44), RV (R^2^ = −0.76), and RN (R^2^ = −0.59) had a strong and positive relationship and negative relationship in RL (R^2^ = −0.69) with GY/ha) (Fig. 2B,C).

#### Yield attributing traits and stress indices

Grain yield in maize is the result of different component traits. It is indirectly calculated by the number of kernels formed in each ear, test weight and number of ears per plant. In I-NDT, a weak relationship for all four traits (C/P, K/R, KR/C, and SW) with GY/ha was observed. However, in WDS-NDT, KR/C (R^2^ = 0.68) had a strong and positive relationship with GY/ha. Similarly, in I-DT, K/R (R^2^ = 0.47) and SW (R^2^ = −0.54) had a strong relationship with GY/ha. However, in WDS-DT, GY/ha was strongly related to most of the traits; for example, a strong and positive relationship was observed in CP (R^2^ = 0.82), and a strong and negative relationship was observed in KR/C (R^2^ = −0.59) and SW (R^2^ = 0.69). Two stress indices (KiSTI and YI) were calculated using the yield data from maize lines under both irrigated and WDS conditions. Thus, a higher and positive R^2^ was expected for all traits (Fig. 1G).

#### SSRTs

The SSRTs were expressed well under WDS conditions. These traits provide a basis for the selection of WDS-tolerant genotypes by a breeder. Eight traits, namely, PM, RLWC, SG, LS, LR, LF, PS, TB and BP, were measured. In I-NDT, BP (R^2^ = −0.42) had a strong and negative relationship with GY/ha, as expected. In WDS-NDT, only SG (R^2^ = 0.67) had a strong and positive relationship with GY/ha. In the case of I-DT, the traits SG (R^2^ = 0.72), LS (R^2^ = 0.96), and LF (R^2^ = 0.62) had a strong and positive relationship with GY/ha, but in PS (R^2^ = −0.43) and TB (R^2^ = −0.44), a strong and negative relationship with GY/ha was observed. In the case of WDS-DT, RLWC (R^2^ = 0.99), SG (R^2^ = 0.99), LF (R^2^ = 0.76), PS (R^2^ = 0.97), and TB (R^2^ = 0.67) had a strong and positive relationship with GY/ha, and LR (R^2^ = 0.99) had a strong and negative relationship with GY/ha (Figs. 1H and 2A).

### Identification of effective phenotypes conferring WDS tolerance in maize

All 38 traits were subjected to principal component analysis (PCA), co-heritability (Co-h^2^) analysis and regression coefficient (R^2^) determination, where GY/ha was kept as a dependent variable. The PCA, Co-h^2^and R^2^ values are presented in Tables 4 and 5 for the irrigated and WDS conditions, respectively. A trait was considered effective if it exhibited high PCA (≥0.20), Co-h^2^ (≥0.60) and R^2^ (≥0.40) values. To investigate the relationships among trait with grain yield (GY/ha) and the factors underlying yield variation, PCA was performed for all the traits. Under irrigated condition PCA explained 46.84% (PCA1: 29.04% and PCA2: 17.80%) in NDT lines and 98.7% variation was explained in DT lines (PCA1: 54.4% and PCA2: 44.3%) for yield variance (Table 4). The traits ASI, DDH, NL, LA-3, LA-C, FLW, EL, H/C, KR, SG, LS, TB, RV and RN were common traits in both NDT and DT lines which had considerable (≥0.20) PCA loading. But, in DT lines some additional traits SU, SL, INL3–4, FLL, EL, EW, C/P, KR/C, SW, RLWC, LR, LF, PS, BP, RFW, RDW and RL had considerable PCA loading. However, under WDS condition PCA explained 47.44% (PCA1: 26.89% and PCA2: 20.55%) in NDT lines and 100% variation was explained in DT lines (PCA1: 56.55% and PCA2: 43.45%) for yield variance (Table 5). The traits DA, DS, NL, LA-C, FLW, EH, EL, KiSTI, LF, PS%, RFW and RDW were common traits in both NDT and DT lines had considerable PCA loading. But, in DT lines some additional traits ASI, SL, INL3–4, FLL, C/P, KR, SG, LS, NBR and RL had considerable PCA loading. The total variance of PCA loading was higher in DT lines under both irrigated and WDS condition. Co-h^2^ with GY/ha was also estimated and all the traits showed high Co-h^2^ except DA, FLL, C/P, LF in NDT and DDH, C/P in DT line under irrigated condition. But, under WDS condition all the traits had high Co-h^2^ in both NDT and DT lines. Co-h2 was higher in WDS condition in comparison to irrigated condition (Table 5).

**Table 4:** Principal component analysis, co-heritability and correlation coefficient estimation of maize inbred lines for thirty-eight traits under irrigated condition.

Traits	NDT				DT			
	PCA-1 (TV = 29.04%)	PCA-2 (TV = 17.80%)	Co-h^2^	R^2^	PCA-1 (TV = 54.4%)	PCA-2 (TV = 44.3%)	Co-h^2^	R^2^
DA	0.23	0.15	0.32	0.00	−0.18	−0.15	0.95	−0.11
DS	0.25	0.11	0.99	0.00	−0.17	−0.16	0.93	−0.07
ASI	0.12	−0.22	0.98	−0.34	0.22	0.03	0.98	0.57
DDH	0.25	0.07	0.98	0.00	−0.05	−0.24	0.09	0.12
PH	0.14	0.23	0.99	0.00	0.15	−0.18	0.99	0.19
NL	0.21	0.11	0.99	−0.22	−0.02	−0.24	0.91	0.17
SU	−0.15	−0.13	0.98	0.02	0.22	0.02	0.99	−0.51
SL	−0.05	−0.19	0.99	−0.02	0.22	0.00	0.99	0.02
LA-3	−0.25	0.06	0.97	0.25	0.07	−0.23	0.99	0.35
LA-C	−0.22	0.10	0.96	0.05	−0.07	−0.23	0.99	0.07
INL 3–4	−0.09	0.13	0.98	0.14	0.20	0.10	0.85	0.27
FLL	−0.04	−0.19	0.46	−0.01	−0.22	0.05	0.97	−0.34
FLW	0.01	−0.32	0.94	−0.18	−0.08	−0.23	0.61	0.04
EH	0.17	0.10	0.99	−0.26	0.01	−0.24	0.93	0.35
EL	0.00	0.32	0.98	0.15	0.22	−0.05	0.98	0.86
EW	−0.11	−0.01	0.87	0.03	0.21	−0.09	0.94	0.96
H/C	−0.25	0.07	0.99	0.27	0.22	0.01	0.81	0.66
C/P	−0.07	0.03	0.43	−0.01	0.13	0.20	0.43	0.01
KR	0.09	0.23	0.99	0.06	0.04	−0.24	0.99	0.47
KR/C	0.17	0.15	0.92	−0.04	0.05	0.24	0.95	−0.14
SW	0.06	0.08	0.96	0.02	−0.22	−0.04	0.85	−0.94
KISTI	−0.17	0.22	0.96	0.87	0.18	−0.14	0.97	0.99
YI	−0.16	0.21	0.96	0.99	0.19	−0.13	0.98	1.00
RLWC	−0.02	0.13	0.95	0.06	0.21	0.09	0.97	0.30
SG	0.11	0.25	0.89	0.01	0.22	−0.01	0.97	0.72
LS	0.15	−0.29	0.91	0.02	0.21	−0.09	0.94	−0.35
LR	0.01	−0.15	0.78	0.17	−0.01	0.24	0.99	0.62
LF	−0.18	−0.04	0.00	0.19	0.22	0.02	0.92	−0.43
PS%	−0.07	−0.16	0.97	−0.42	−0.22	−0.06	0.83	0.00
TB	−0.23	−0.03	0.98	0.01	−0.21	0.09	0.89	−0.44
BP	0.05	−0.19	0.98	−0.42	0.13	0.20	0.82	0.00
NBR	0.26	−0.02	0.97	−0.30	0.16	−0.17	0.96	0.96
RFW	0.18	0.04	0.81	−0.07	−0.09	−0.22	0.91	0.04
RDW	0.20	−0.06	0.94	−0.21	0.03	−0.24	0.99	0.44
RL	0.16	−0.09	0.93	0.02	−0.08	0.22	0.93	−0.69
RV	0.18	−0.25	0.96	−0.69	0.22	−0.02	0.94	0.76
RN	0.27	0.01	0.98	−0.19	0.06	−0.23	0.98	0.59

PCA: Principal component analysis; Co-h2: Co-heritability; r2: regression coefficient; TV = total variance explained; DA: Days to 50% tasseling; DS: Days to 50% silking; ASI: Anthesis silk interval; DDH: Days to 75% dry husk; PH: Plant height; NL: Number of leaves per plant; SU: Number of stomata on upper surface of leaf; SL: Number of stomata on lower surface of leaf; LA-3: 3rd leaf angle from top; LA-C: Leaf angle at ear forming node; INL3–4: Internodal length between 3rd and 4th leaf from top; FLL: Flag leaf length; FLW: Flag leaf width; EH: Ear height; EL: Ear length; EW: Ear width; H/C: Number of husk per ear; C/P: Number of ear per plant; K/R: Number of kernels per rows; KR/C: Number of kernel rows per ear; SW: 1000 seed weight; GY/ha: Grain yield per hectare; KiSTI: Modified stress tolerance index; YI: Yield index; RLWC: Relative leaf water content; SG: Stay green; LS: Leaf senescence; LR: Leaf rolling; LF: Leaf firing; PS%: Pollen stainability; TB: Tassel blast and BP: Bareness percentage; NBR: Number of brace root per plant; RFW: Root fresh weight; RDW: Root dry weight; RL: Root length; RV: Root volume; RN: Number of roots >10 cm.

**Table 5:** Principal component analysis, co-heritability and correlation coefficient estimation of maize inbred lines for thirty-eight traits under WDS condition.

Traits	NDT				DT			
	PCA-1 (TV = 26.89)	PCA-2 (TV = 20.55)	Co-h^2^	R^2^	PCA-1 (TV = 56.56)	PCA-2 (TV = 43.46)	Co-h^2^	R^2^
DA	0.26	0.15	0.96	0.53	0.20	−0.09	0.97	−0.13
DS	0.27	0.08	0.97	0.43	0.21	−0.08	0.93	−0.02
ASI	0.19	−0.06	0.93	0.02	−0.01	0.25	0.98	0.4
DDH	0.26	0.06	0.95	0.33	0.19	−0.12	0.97	−0.76
PH	0.22	0.10	0.99	0.93	0.16	0.17	0.99	0.55
NL	0.28	−0.10	0.93	0.22	0.01	−0.25	0.98	0.48
SU	0.02	−0.13	0.91	0.61	−0.16	−0.17	0.99	−0.43
SL	0.04	0.18	0.99	0.69	−0.09	−0.23	0.96	−0.79
LA-3	−0.12	0.21	0.94	0.60	−0.18	−0.15	0.93	−0.59
LA-C	−0.21	0.04	0.97	0.26	−0.02	−0.25	0.82	−0.98
INL 3–4	−0.06	0.09	0.97	−0.02	−0.06	0.24	0.99	0.99
FLL	−0.02	−0.13	0.93	−0.87	−0.21	0.08	0.98	0.60
FLW	−0.16	−0.26	0.96	−0.35	0.22	0.03	0.94	0.99
EH	0.27	0.02	0.92	0.17	0.13	−0.20	0.98	0.01
EL	0.09	0.20	0.99	0.49	0.22	0.01	0.97	0.56
EW	0.11	0.25	0.90	0.01	0.18	−0.15	0.95	−0.01
H/ C	−0.08	0.19	0.92	0.38	−0.14	−0.19	0.98	−0.89
C/ P	−0.06	0.07	0.98	−0.17	0.10	0.22	0.98	0.82
KR	0.09	0.14	0.91	0.21	0.21	−0.07	0.95	0.07
KR/C	0.20	0.21	0.97	0.68	0.15	−0.18	0.96	−0.59
SW	−0.12	0.17	0.94	0.25	0.19	−0.11	0.90	−0.69
KISTI	0.05	0.28	0.99	0.85	0.12	0.21	0.96	0.98
YI	0.04	0.31	0.95	0.74	0.14	0.19	0.99	0.99
RLWC	0.05	0.16	0.94	0.01	0.19	0.12	0.97	0.99
SG	0.03	0.17	0.90	0.67	0.12	0.21	0.99	0.96
LS	−0.06	−0.07	0.92	0.04	−0.20	−0.11	0.99	0.99
LR	0.05	−0.28	0.99	0.01	−0.17	−0.15	0.97	0.76
LF	−0.21	0.14	0.93	0.10	−0.09	0.23	0.97	−0.44
PS%	0.29	0.03	0.94	−0.27	0.22	−0.03	0.98	−0.64
TB	−0.21	−0.08	0.95	0.05	0.16	−0.17	0.99	−0.67
BP	−0.06	−0.13	0.98	−0.27	−0.19	−0.12	0.99	−0.64
NBR	0.09	−0.08	0.92	−0.06	0.10	−0.22	0.98	0.01
RFW	0.19	−0.21	0.93	0.01	0.20	−0.09	0.99	0.04
RDW	0.20	−0.20	0.98	0.02	0.20	−0.09	0.92	0.01
RL	0.09	−0.03	0.09	0.31	0.21	−0.07	0.93	0.01
RV	0.18	−0.21	0.92	−0.13	0.10	0.22	0.90	−0.98
RN	0.24	−0.17	0.89	0.08	0.22	−0.03	0.91	−0.31

PCA: Principal component analysis; Co-h^2^: Co-heritability; r^2^: regression coefficient; TV = total variance explained; DA: Days to 50% tasseling; DS: Days to 50% silking; ASI: Anthesis silk interval; DDH: Days to 75% dry husk; PH: Plant height; NL: Number of leaves per plant; SU: Number of stomata on upper surface of leaf; SL: Number of stomata on lower surface of leaf; LA-3: 3^rd^ leaf angle from top; LA-C: Leaf angle at ear forming node; INL3–4: Internodal length between 3^rd^ and 4^th^ leaf from top; FLL: Flag leaf length; FLW: Flag leaf width; EH: Ear height; EL: Ear length; EW: Ear width; H/C: Number of husk per ear; C/P: Number of ear per plant; K/R: Number of kernels per rows; KR/C: Number of kernel rows per ear; SW: 1000 seed weight; GY/ha: Grain yield per hectare; KiSTI: Modified stress tolerance index; YI: Yield index; RLWC: Relative leaf water content; SG: Stay green; LS: Leaf senescence; LR: Leaf rolling; LF: Leaf firing; PS%: Pollen stainability; TB: Tassel blast and BP: Bareness percentage; NBR: Number of brace root per plant; RFW: Root fresh weight; RDW: Root dry weight; RL: Root length; RV: Root volume; RN: Number of roots >10 cm.

## Discussion

### Rainfall pattern

Maize is an extremely water-sensitive crop and most of the maize-grown areas are rain-fed. Therefore, maize in India faces WDS, which is detrimental to maize production. It is a well-accepted crop by farmers of eastern India, but the intensity of intermittent stress determines its production. In eastern India, the rainfall variability and frequency of water shortages are high, and the crop faces WDS in both the wet and dry seasons. The weather data indicated a higher frequency of WDS from the months of August to September in the wet season and after the 1^st^ fortnight of February in the dry season (Supplementary Fig. 1). Usually, crops are at the flowering and grain-filling stages in these months if they are sown in a timely manner. The intensity of WDS under field conditions is random in each season and even in each week of crop growth. Hence, sometimes known WDS-tolerant lines may fail to perform well under actual field conditions. This failure occurs because the experimental conditions were not truly representative of farmers’ fields and WDS-tolerant lines perform best under a constant magnitude of stress. Thus, we created variation in WDS approximating the prevalent WDS conditions of eastern India.

### Variation in the performance of maize lines

The phenotypic expression of all 38 traits was statistically tested using a t-test. We observed that all the traits were significantly differentially expressed between irrigated and WDS conditions (Tables 2 and 3). Even significant phenotypes differences were also seen between the NDT and DT maize lines under stress for maximum number of traits (Supplementary Table 1). This variation was because of differences in the genetic constitution of the lines, which depends on the variability in the source populations from which the lines were obtained and developed^23,24^. Accordingly, the maximum variation among the lines was captured to identify the potential traits associated with WDS tolerance.

In general, maize is more susceptible to WDS than other rain-fed cereal crops because of its geitonogamous nature. Many traits attain a higher percentage of expression (high mean) under WDS condition in both types of maize lines i.e., DA (by 9.81% in NDT and 10.10% in DT), DS (by 14.33% in NDT and 14.68% in DT), ASI (by 86.44% in NDT and 115.80% in NDT) and DDH (by 11.98% in NDT and 10.60% in DT), LS (by score of 2.21 in NDT and 2.39 in DT), LR (by score of 3.93 in NDT and 3.05 in DT), LF (by score of 1.82 in NDT and 2.09 in DT), TB (by score of 3.42 in NDT and 3.37 in DT) and BP (by 21.88% in NDT and 25.37% in DT). As a consequence WDS delayed flowering (DA), delayed silk extrusion (DS) and resulted in asynchrony between pollen dehiscence and female receptivity (ASI)^19,25–27^. Therefore, it promoted a longer ASI. A delay in DS encourage poor fertilization which results in a higher BP^28^ and higher rate of floral abortion decreases the seed set in the ear^29,30^. A negative change in the leaf water potential of lines increases the internal leaf temperature to save internal water, indicating phenotypic adaptation to a higher LR. Gradually, the leaf internal temperature rises and combines with the higher air temperature to induce a higher TB (by a score up to 4.90) and LF (by a score up to 2.60) in the top leaves, under WDS^31^. Simultaneously, higher expression of LS (by a score up to 4.89) occurs due to the degradation of chlorophyll in the photosynthetic apparatus^32–34^. Overall, these negative changes in leaf performance cause a loss of the normal phenotype and affect yield^13^.

The remaining phenological traits displayed a lower mean value under WDS in both types of maize lines. The reduction in PH (by 18.24% in NDT and 19.22% in DT) under WDS^16,35^ is attributed to a decline in cell enlargement and higher LS^18^. Generally, maize plants produce 13 numbers of leaves, one at each internode, after which the tassel appears. In the present experiment averages of 12 numbers of leaves were formed in maize lines under irrigated conditions. The increase in the mean of LR, LS and LF under WDS promotes lower the resource capture, lower canopy photosynthesis; as a consequence, the PH and NL were reduced to an average of 11^17^. This effect extended to leaf size FLL (by 12.23% in NDT and 28.28% in DT) and FLW (by 18.00% in NDT and 31.75% in DT), which also decreased^18^ under WDS. The leaf stomata (SL and SU) played a role in internal water and temperature regulation of the plants. The SL was invariably higher in number than SU but, overall their number was reduced due to WDS on both surfaces of the leaves viz; SL by 10.58% in NDT and 8.00% in DT); SU by 11.95% in NDT and 10.09% in DT. The stomatal conductance decreased, which has a direct proportional relationship with yield^36^. In maize, the flag leaf and the 2^nd^ leaf from the top are much smaller than the 3^rd^ leaf from the top (LA-3). The amount of light interception in a crop area depends on these leaf angles. LA-3 and INL3–4 determine light penetration, and LA-C indirectly measures the ear angle. Due to the greater leaf angle of the lower leaves and the smaller leaf angle of the upper leaves, light penetration and canopy photosynthesis increase^37^. Modern maize cultivars have such phenotypes and produce a higher yield^38^. Photosynthesis, which affects yield, largely takes place in five or six leaves near and above the ear^39^. We observed a 25–30° leaf angle, which supports higher light utilization by plants and provides easy passage of pollen to the stigma.

Photosynthates and carbohydrates are translocated to grains through the ear, where H/C serves as a reservoir of carbohydrates for supply during grain filling and maintains a high water content to protect the grains from abnormal external temperature and bird damage^24^. The mean value of the ear traits such as EH (by 19.08% in NDT and 26.04% in DT), EL (by 36.07% in NDT and 30.03% in DT), EW (by 27.68% in NDT and 32.30% in DT) and H/C (by 6.16% in NDT and 0.12% in DT)] was reduced under WDS. Lower EH sometimes considered as preferred because it aids to the development of short-statured plants, which leads to less lodging, but below 1 m height may lead to animal damage. EL and EW were also lower under WDS^40^ as a consequence of a smaller number of grains than under normal conditions.

The effect of WDS on expression of yield and stress indices was decrease for C/P (by 18.87% in NDT and 16.51% in DT), K/R (by 36.72% in NDT and 29.91% by DT), KR/C (by 29.55% in NDT and 29.69% in DT), SW (by 8.86% in NDT and 17.67% in DT), GY/ha (by 51.49% in NDT and 48.55% in DT), KiSTI (by 16.67% in NDT and 2.63% in DT), and YI (by 12.96% in NDT and 3.33% in DT). The reduction in C/P was attributed due to a reduction in PH and increase in LS, which restricted further development of C/P. As a result, less than one C/P was formed under WDS. The traits K/R, KR/C and SW were also decreased under WDS due to embryo abortion, delay in the appearance of DS and a shortage in the carbohydrate reserve under WDS^41^. The stress index for some genotypes was lower and some had higher values. The highest value was observed for the WDS-tolerant genotypes. These genotypes can sustain a good yield under WDS in comparison to irrigated conditions^42^. The SSRT group of traits displayed both increased and decreased in expression under WDS. Some traits, such as LS, LR, LF, TB and BP showed higher expression, and others such as RLWC (by 15.36% in NDT and 19.90% in DT), SG (by 2.28% in NDT and 31.82% in DT) and PS (by 52.58% in NDT and 52.73% in DT) had lower expression under WDS. The reduction in RLWC is attributed to a reduction in the performance of leaf traits. Similarly, SG was also reduced because of a reduction in the performance of leaf traits and the higher expression of LS and LF. Furthermore, PS was lower under WDS because of disturbance of meiosis and carbohydrate metabolism^43,44^.

The imposed stress suppressed the below ground traits expression such as RFW (by 38.87% in NDT and 50.00% in DT), RDW (by 26.01% in NDT and 50.74% in DT), RL (by 39.79% in NDT and 45.17% in DT), RV (by 43.14% in NDT and 26.67% in DT) and RN (by 14.16% in NDT and 30.62% in DT). The trait NBR also reduces under WDS by 51.06% in NDT and 46.09% in DT maize lines. Under slight WDS, root traits are reportedly enhanced in the search for water^45^. However, under high stress intensity, the overall root architecture development is hampered and thus results in a abnormal root pattern. A reduction in overall plant growth; an increase in flowering, days to maturity and ASI; a reduction in mean vegetative, leaf, and ear traits; abnormal expression of SSRTs; and a loss of normal root architecture lead to a reduction of overall GY/ha^24,46,47^.

### Comparison between DT and NDT lines under WDS

The change due to WDS in the performance of NDT and DT lines was compared for all 38 traits (Supplementary Table 2). DT maize lines tend to exhibit early flowering and maturity i.e, DA (0.13%), DS (1.29%), DDH (10.09%) and a lower ASI (3.41%) under WDS than NDT lines. ASI under drought has become shorter in modern cultivars, and the selection of such individuals increases the growth of ears^48^. The DT lines that exhibited high PH (7.72%), NL (7.95%), LA-C (43.23%), FLL (149.85%), EH (44.27), EW (13.87%), RL (27.34%), SW (118.30%), GY/ha (4.29%), RLWC (21.42%), SG (61.71%), RFW (18.79%), RDW (114.67%), RL (27.34%), RN (118.96%), and suitably lower LR (22.39%) and TB (1.46%) ultimately added a higher GY/ha under WDS than NDT lines. However, higher expression of LS (8.14%), LF (15.47%) and BP (15.95%) in DT lines is not desirable, and these traits were less expressed in the NDT lines. Some traits (such as SL, FLL, FLW, EW, RFW, RDW, RN, KR/C and RLWC) exhibited greater expression in the NDT lines than in the DT lines. The variation in phenotypes between NDT and DT lines is genetic since both were evaluated under alike environmental conditions. The DT lines displayed some favorable or sustainable trait expression under WDS, which contribute yield under WDS. These findings were also reported in previous research^49^. The traits that are distinct in DT and NDT lines are leaf area, C/P, PS, SG, EL^24^, KiSTI, YI^42^, NL, PH, SU (stomatal conductance), RL, RV^50^, SW^49^, LS, ASI^28^, and LR^51^.

Overall, a greater (≥30% and a score of ≥2) reduction was observed for traits such as ASI, EL, K/R, GY/ha, NBR, RFW, RL, RV, PS, LS, LR and TB in the NDT lines. Similarly, in the DT lines, ASI, FLW, EL, EW, GY/ha, NBR, RFW, RDW, RL, RN, SG, PS, LS, LR, LF and TB exhibited a greater reduction (Tables 2 and 3). Thus, the above 12 traits in the NDT lines and 16 traits in the DT lines were more sensitive to WDS than other traits. Thus, it is apparent that DT lines had different expression pattern for above traits which provide higher buffering capacity under WDS.

### Relationship between grain yield and phenological traits

Breeding in maize primarily concerns yield improvement under target environment. Proper growth and development of maize plants comprise numerous parameters that are estimated by different traits. To understand the behavior of the traits in our present experiments, all the traits were plotted in linear regression curves against GY/ha. The coefficient of determination (R^2^) was large (>40%), and a high percentage of the yield variation was explained by KiSTI (87%), YI (99%), BP (−42%) and RV (−69%) in I-NDT. However, in WDS-NDT, a large R^2^ was obtained, and a high percentage of variation in yield was explained by DA (53%), DS (43%), PH (93%), SU (61%), SL (69%), LA-3 (60%), FLL (−87%), EL (49%), KR/C (68%), KiSTI (85%), YI (74%) and SG (67%). Similarly, for the I-DT lines, a high percentage of yield variation was explained by ASI (57%), NL (51%), SU (−51%), EL (86%), EW (96%), H/C (66%), K/R (47%), SW (−94%), KiSTI (99%), YI (99%), SG (72%), LR (62%), LF (−43%), TB (−44%), NBR (96%), RDW (44%), RL (−69%), RV (76%), and RN (59%). However, in the WDS-DT lines, a large R^2^ was obtained, and a high percentage of variation in yield was explained by all the traits except for 10 traits (DA, DS, EH, EW, K/R, NBR, RFW, RDW, RL and RN). The association between phenological traits and GY/ha had stronger relation under WDS conditions than under irrigated conditions, which shows worthiness of the measure traits. The traits RV, BP, KiSTI and YI were similar variation under irrigated conditions for the NDT and DT lines. Similarly, the traits SG, KR/C, EL, FLL, LA-3, SL, SU, PH, RV, BP, KiSTI and YI were similar variation under WDS for the NDT and DT lines. Researchers have also reported a strong relationship of yield with kernel traits^28^, flowering and ASI^26^, C/P^24^, EL and EW^52^, and SW and NL^53^. However, for many of the traits, such a relationship has not been reported, but an association of grain yield with other traits such as DA, DDH^54^, leaf traits, leaf angle, SG, BP, LR, and TB^55^ has been reported. Thus, using these traits, it is possible to predict the yield of a line.

### Identification of effective phenotypes conferring WDS tolerance

Many phenotypic traits lead to a higher buffering capacity in maize lines in adverse environments^13^. The association of such traits with yield-related traits enhances the potential yield of lines. Generally, we used single statistics to screen relevant traits but ignored some other relationships, such as the heritability of the traits and their relationships with yield. Thus, multiple statistical analyses were performed for precise selection of traits in the present experiment. Multivariate statistics by PCA was performed to identify traits with greater contribution towards yield variance. In NDT lines PCA explained a lower variance (Irrigated: 46.84% and WDS: 47.44%) compared to DT lines (Irrigated: 98.7% and WDS: 100%) for yield. Thus, traits expression in DT line more closely related to yield variance and under WDS the relation was stronger. Beside, some common traits (both NDT and DT) showed higher loading in PCA, there were additional traits in DT lines which had higher loading (ASI, NL, SL, LA-C, INL3-4, FLL, FLW, KR, C/P, SG, LS, LS, PS%, NBR, RL, RDW and RL) towards yield variance under WDS condition. These traits also had high Co-h^2^, which is more desirable for selection under WDS. Using a combination of PCA, Co-h^2^, and R^2^, we found traits in the DT lines that were closely related to yield in both environments. Furthermore, the phenotypic expression of DT lines was more prominent than that of NDT lines, especially under WDS. Thus, relevant and effective traits were chosen from DT lines: NL, SL, LA-C, INL3-4, FLL, FLW, C/P, LS, PS%, RDW and RL. Some traits such as kernel set, grain yield, ASI, silk emergence, ear formation, ear size (or ear growth rate), adequacy of pollen viability, ears per plant, barrenness, kernels per ear, weight per kernel, and stay-green have also been associated with WDS tolerance^24,27^. These valuable traits must be combined with phenotypic selection for WDS tolerance in maize to construct a proper plant ideotype rather than selecting by only yield per se. Such a use of multiple trait selection has also been previously reported^12^. Multiple selection criteria were also previously used^48^ to obtain a higher yield per breeding cycle, in which different traits were chosen based on their variance, heritability and genetic correlation with yield, and recently, eigenvalues (principal components) were also used^56,57^ obtained higher yield gains under severe moisture stress conditions in maize by using a selection index.

A trait was considered effective if it exhibited high PCA (≥0.20), Co-h^2^ (≥0.60) and R^2^ (≥0.40) values. To investigate the relationships among trait with grain yield (GY/ha) and the factors underlying yield variation, PCA was performed for all the traits. Under irrigated condition PCA explained 46.84% (PCA1: 29.04% and PCA2: 17.80%) in NDT lines and 98.7% variation was explained in DT lines (PCA1: 54.4% and PCA2: 44.3%) for yield variance (Table 4). The traits ASI, DDH, NL, LA-3, LA-C, FLW, EL, H/C, KR, SG, LS, TB, RV and RN were common traits in both NDT and DT lines which had considerable (≥0.20) PCA loading. But, in DT lines some additional traits SU, SL, INL3-4, FLL, EL, EW, C/P, KR/C, SW, RLWC, LR, LF, PS, BP, RFW, RDW and RL had considerable PCA loading. However, under WDS condition PCA explained 47.44% (PCA1: 26.89% and PCA2: 20.55%) in NDT lines and 100% variation was explained in DT lines (PCA1: 56.55% and PCA2: 43.45%) for yield variance (Table 5). The traits DA, DS, NL, LA-C, FLW, EH, EL, KiSTI, LF, PS%, RFW and RDW were common traits in both NDT and DT lines had considerable PCA loading. But, in DT lines some additional traits ASI, SL, INL3-4, FLL, C/P, KR, SG, LS, NBR and RL had considerable PCA loading. The total variance of PCA loading was higher in DT lines under both irrigated and WDS condition. Co-h^2^ with GY/ha was also estimated and all the traits showed high Co-h^2^ except DA, FLL, C/P, LF in NDT and DDH, C/P in DT line under irrigated condition. But, under WDS condition all the traits had high Co-h^2^ in both NDT and DT lines. Co-h2 was higher in WDS condition in comparison to irrigated condition. Considering the PCA, Co-h^2^, and R^2^ together, the most effective traits under irrigated conditions were KiSTI, YI, NBR, and RV for the NDT lines and ASI, SU, EL, EW, H/C, KR, SW, SG, LR, LF, TB, RDW, RL, and RV for the DT lines (Table 4).

## Conclusion

An exposure of WDS at flowering and grain filling stage brought severe negative effects on phenological and yield traits attributes of the maize lines. Concurrently the performance of NDT and DT maize lines differed for several traits under WDS. The mean value of traits was below the desirable limit but certain genotypes showed higher mean under WDS due to their different genetic background and buffering capacity. WDS at flowering and grain filling stage leads to significant yield penalty especially in NDT lines than DT lines. The traits viz; NL, SL, LA-C, INL3-4, FLL, FLW, C/P, LS, PS%, RDW and RL were identifies specific to improve WDS tolerance in maize. In such context, the WDS tolerance traits should be in plant ideotype while selecting a line in breeding. The maize lines showed highly desirable phenotypic expression under WDS for any traits are important to conserve to identify novel gene.

## Supplementary information

Comparison of weather variable of last 25–30 years with weather of years of experimentation.

Dataset 1.

Dataset 2.

## Data Availability

All data analyzed during this study are included in this published article in Supplementary Table 1.
