# Supplementary material for: Impact of water deficit stress in maize: Phenology and yield components
Source: Sci Rep. 2020 Feb 19;10:2944. doi: 10.1038/s41598-020-59689-7 (PMC7031221; doi:10.1038/s41598-020-59689-7)
Supplement: Supplementary file 1 — Comparison of weather variable of last 25–30 years with weather of years of experimentation. [file 41598_2020_59689_MOESM1_ESM.pdf]

Impact of water deficit stress in maize: Phenology and yield components

R. P. Sah[a+], M. Chakraborty*[b], K. Prasad[c], M. Pandit[d], V. K. Tudu[d], M. K. Chakravarty[e], S. C. Narayan[d], M. Rana[f], and D. Moharana[g]

[a]Presently: Scientist, ICAR-National Rice Research Institute, Cuttack, Odisha, India.

[+]Ph.D. scholar, Department of Genetics and Plant Breeding, Birsa Agricultural University, Kanke, Ranchi, Jharkhand, India.

[b]Chief Scientist, Department of Genetics and Plant Breeding, Birsa Agricultural University, Kanke, Ranchi, Jharkhand, India.

[c]Scientist, Department of Genetics and Plant Breeding, Birsa Agricultural University, Kanke, Ranchi, Jharkhand, India.

[d]Research Scholar, Department of Genetics and Plant Breeding, Birsa Agricultural University, Kanke, Ranchi, Jharkhand, India.

[e]Chief Scientist, Department of Entomology, Birsa Agricultural University, Kanke, Ranchi, Jharkhand, India

[f]Scientist, Indian Grassland and Fodder Research Institute, Jhansi, Uttar Pradesh, India

[g]Research Scholar, ICAR-National Rice Research Institute, Cuttack, Odisha, India.

***Corresponding author:**

Dr. Manigopa Chakraborty

Chief Scientist-Cum University Professor, Birsa Agricultural University, Kanke, Ranchi, Jharkhand, India

[*]Corresponding author: manigopa291061@yahoo.com

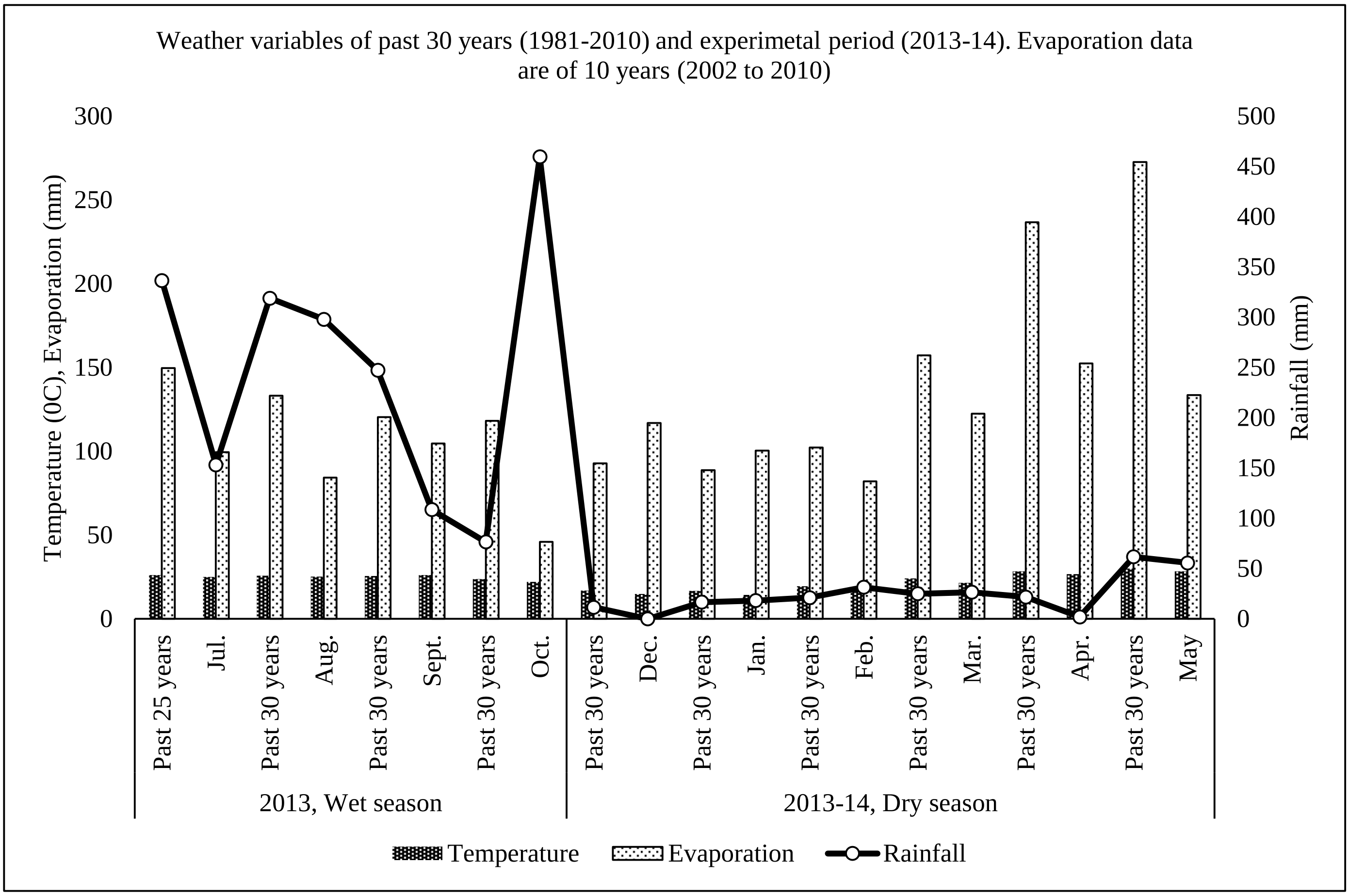

Supplementary Fig. 1. Comparison of weather variable of last 25-30 years with weather of years of experimentation
